# Desymmetrization of 7-azabicycloalkenes by tandem olefin metathesis for the preparation of natural product scaffolds

**DOI:** 10.1186/1860-5397-3-48

**Published:** 2007-12-18

**Authors:** Wolfgang Maison, Marina Büchert, Nina Deppermann

**Affiliations:** 1 Institut für Organische Chemie, Heinrich-Buff-Ring 58, Justus-Liebig-Universität Gießen, 35392 Gießen, Germany

## Abstract

**Background:**

Tandem olefin metathesis sequences are known to be versatile for the generation of natural product scaffolds and have also been used for ring opening of strained carbo- and heterocycles. In this paper we demonstrate the potential of these reactions for the desymmetrization of 7-azabicycloalkenes.

**Results:**

We have established efficient protocols for the desymmetrization of different 7-azabicycloalkenes by intra- and intermolecular tandem metathesis sequences with ruthenium based catalysts.

**Conclusion:**

Desymmetrization of 7-azabicycloalkenes by olefin metathesis is an efficient process for the preparation of common natural product scaffolds such as pyrrolidines, indolizidines and isoindoles.

## Background

Azabicyclo [x.y.0]alkane scaffolds are ubiquitous structural elements in pharmaceutically important peptide mimetics [1–3] and several important classes of natural products such as indolizidine and quinolizidine alkaloids and azasugars. [4–6] In consequence, a number of groups have developed efficient syntheses of these bicyclic heterocycles. [7–9] Challenges for the synthesis of these structures are the introduction of chirality and of several functional groups into the scaffolds. In particular the latter point is often a problem, leading to multistep sequences.

**Figure 1:**
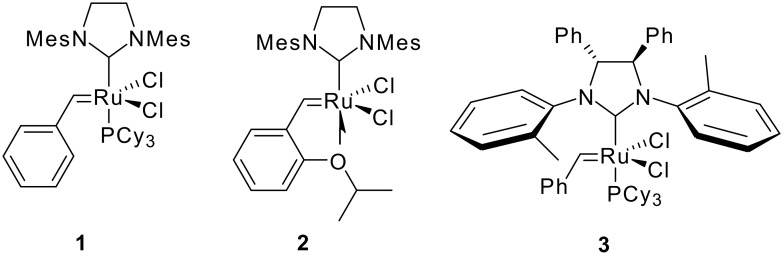
Ruthenium based precatalysts used in this study.

In this context, ring closing metathesis (RCM) and tandem metatheses [10–13] have been particularly successful strategies for the assembly of common natural product scaffolds. [14–22] A general advantage of these approaches is that ring closure and/or scaffold-rearrangements can be accomplished while generating a double bond as a valuable functional group for further manipulations. In addition, the common ruthenium (Figure 1) and molybdenum based catalysts for olefin metathesis are well known for their broad functional group tolerance.

The application of RCM to the synthesis of azabicycloalkane scaffolds was first described by Grubbs[23] for the synthesis of peptide mimetics and later extended by several other groups. [24–31] Key intermediates in these approaches are often alkenyl substituted pyrrolidines, which are *N*-acylated with an unsaturated carboxylic acid and submitted to a ring closing metathesis (RCM).

**Scheme 1:**
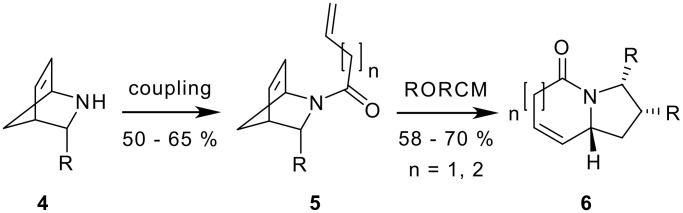
RORCM of 2-azabicycloalkenes **5** to bicyclic scaffolds **6**.

As a part of a general synthetic concept using azabicycloalkenes as masked analogs of functionalized pyrrolidines or piperidines [32–39] we have previously applied the concept of intramolecular ring-opening/ring-closing metathesis (RORCM) [40–54] to *N*-acylated 2-azabicycloalkenes **4** as precursors for azabicyclo [X.3.0]alkanes like **6** (Scheme 1).[55] Various other strained heterocycles have also been used for ring opening metathesis or other tandem metathesis sequences. [56–61]

In this paper, we describe the extension of this work and show that desymmetrization of 7-azabicycloalkenes *via* RORCM leads to valuable natural product scaffolds. In this context, symmetrical derivatives of 7-azabicycloalkenes like **7** and **12** are extremely interesting substrates for RORCM conversions, because they may be desymmetrized either by diastereoselective or enantioselective metathesis.

## Results and Discussion

In a first attempt to transfer the RORCM-strategy to 7-azabicycloalkenes, we chose **7** as a precursor for domino metathesis reactions. Our choice was due to the following two reasons: 1. Azabicycloalkene **7** is easy to synthesize *via* Diels-Alder reaction.[62] 2. It was assumed to be a good substrate for RORCM because it is strained and has been shown to be susceptible to other desymmetrizing ring opening reactions in the past.[63–64]

To generate appropriate precursors for the tandem conversions, **7** was deprotected and acylated with butenoic acid and pentenoyl chloride to give **8** and **10**. However, first attempts to convert the bis-olefin **8** *via* RORCM to the bicyclic target structure failed and only pyridone derivative **9** was isolated in small quantities along with large amounts of unreacted starting material. With turnover numbers of only three, the ruthenium based catalysts **1** and **2** were both quite ineffective in this metathesis reaction.

**Scheme 2:**
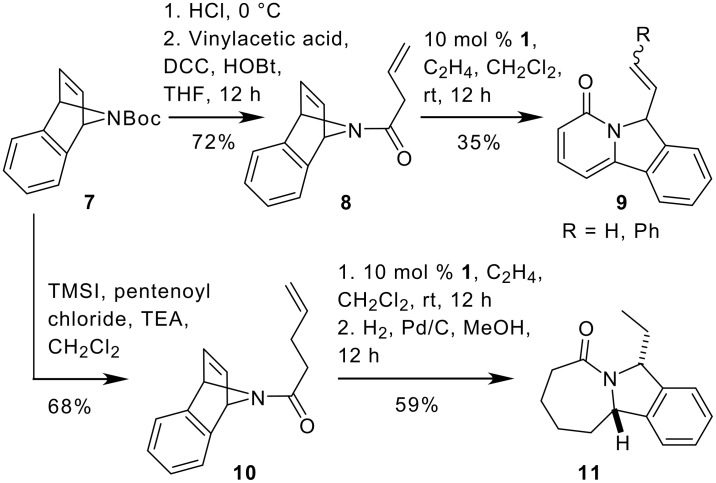
RORCM of 7-azabicycloalkenes **8** and **10** to pyridone **9** and isoindole scaffold **11**.

We assumed that the structure of the starting material **8** (location of the exocyclic double bond) and the following aromatization to **9** was the reason for the low catalytic efficiency of this conversion and tested this hypothesis with the conversion of the corresponding pentenoyl derivative **10** under RORCM conditions. As outline in Scheme 2, this reaction gave the expected metathesis product, which was hydrogenated to the isoindole derivative **11** in good yield, verifying our previous assumptions.

As a general trend, it turned out that benzannelated azabicycloalkene derivatives like **8** and **10** give relatively unstable products. In consequence, products can only be isolated as pyridones **9**, derived from spontaneous aromatization or have to be hydrogenated to their saturated analogues **11**.

**Scheme 3:**
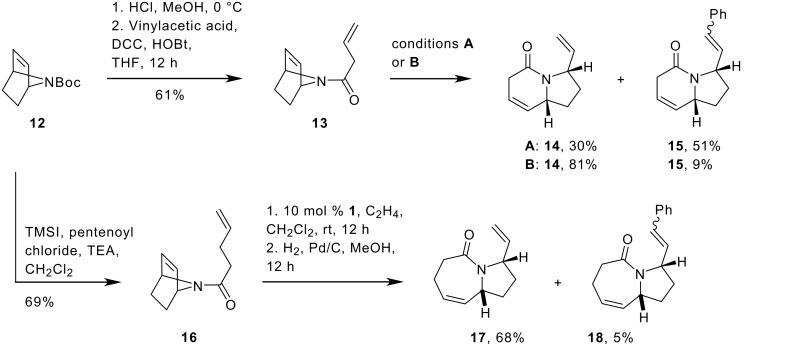
RORCM of 7-azabicycloalkenes **13** and **16** to bicyclic scaffolds **14**, **15**, **17** and **18**. Conditions **A**: 10 mol % **1**, C_2_H_4_, 3 equiv styrene, CH_2_Cl_2_, rt, 12 h; **B**: 10 mol % **1**, C_2_H_4_, CH_2_Cl_2_, rt, 12 h.

This unique reactivity of benzannelated metathesis precursors like **8** and **10** is not observed with other 7-azabicycloalkenes like **13** and **16** as depicted in Scheme 3. Starting from the known Boc-protected heterocycle **12**,[65–66] RORCM precursors **13** and **16** were generated after deprotection under standard acylation conditions in good yields. Treatment of these bis-olefins with Grubbs catalyst **1** gave the expected bicyclic compounds **14** and **17** in good yield along with some byproducts **15** and **18**, respectively. These byproducts are often observed, if the generated exocyclic double bond in the RORCM product is susceptible to olefin cross metathesis with the small amount of styrene that is derived from the precatalyst **1**. These types of products are favored, if additional olefins are added as CM partners. With addition of 3 equivalents styrene (condition **A** in Scheme 3), for example, the styrene adduct **15** becomes the main product.

**Scheme 4:**
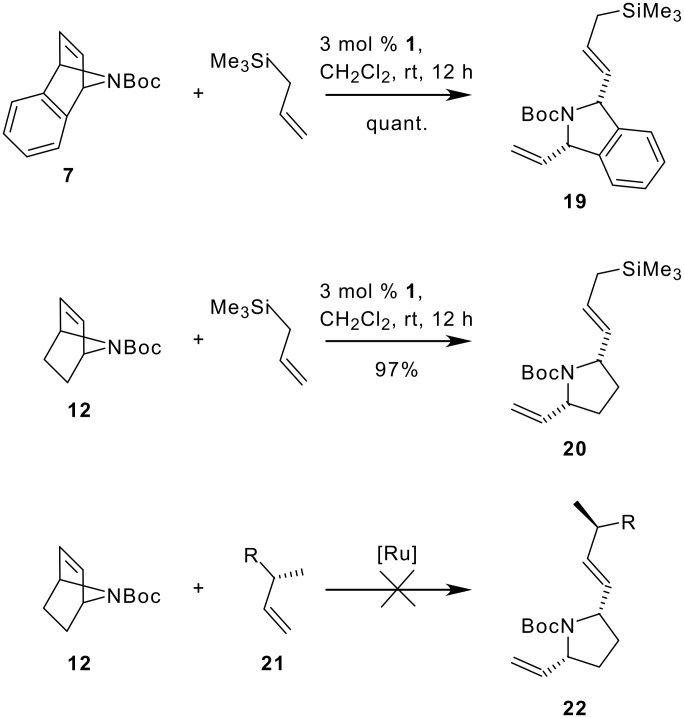
ROCM of 7-azabicycloalkenes **7** and **12** to isoindole and pyrrolidine scaffolds **19** and **20**.

Having established suitable protocols for conversions of 7-azabicycloalkenes to racemic products, we tried next to develop stereoselective variants and started our studies again with Boc-protected 7-azabicycloalkenes **7** and **12**. A sequence of ring opening and cross metathesis is extremely efficient for desymmetrization of **7** and **12** as depicted in Scheme 4 for the synthesis of isoindole **19** and the disubstituted pyrrolidine **20**. In these cases, catalyst loadings can be low and yields are excellent.

Unfortunately the ROCM of 7-azabicycloalkenes appeared to be quite sensitive with respect to the olefin cross metathesis partner [67] and we have not been able to transfer this reaction to α-substituted olefins like **21** yet.

**Scheme 5:**
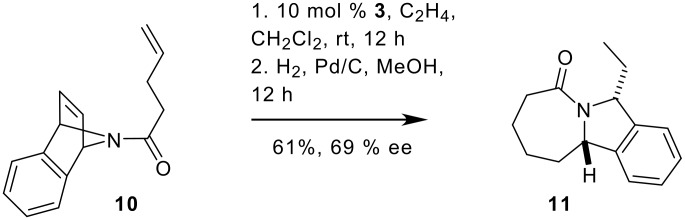
Catalytic enantioselective desymmetrization of 7-azabicycloalkene **10** to scaffold **23**.

A more successful attempt to introduce selectivity, was the enantioselective catalytic desymmetrization of bis-olefin **10** with the known chiral ruthenium catalyst **3**.[67] This reaction gave enantioenriched **11** in good yield (Scheme 5). However, the enantioselectivity of this reaction is only moderate compared to similar reactions using molybdenum based precatalysts and different azabicycloalkene starting materials that have been recently reported by Hoveyda and Schrock for the enantioselective preparation of piperidines.[68–69]

## Conclusion

In this paper we have described efficient tandem metathesis protocols for the desymmetrization of 7-azabicycloalkenes. Desymmetrization is accomplished by intramolecular RORCM or intermolecular ROCM sequences to give a range of common natural product scaffolds such as pyrrolidines, indolizidines and isoindoles. The protocols use readily available starting materials, are simple and give densely functionalized metathesis products ready for further manipulations.

## Supporting Information

File 1 Experimental. Experimental procedures for the synthesis of all compounds described, and characterization data for the synthesized compounds.
