# Supplementary material for: Desymmetrization of 7-azabicycloalkenes by tandem olefin metathesis for the preparation of natural product scaffolds
Source: Beilstein J Org Chem. 2007 Dec 18;3:48. doi: 10.1186/1860-5397-3-48 (PMC2200670; doi:10.1186/1860-5397-3-48)
Supplement: File 1 — Experimental. Experimental procedures for the synthesis of all compounds described, and characterization data for the synthesized compounds. [file Beilstein_J_Org_Chem-03-48-s001.doc]

**Experimental**

**Desymmetrization of 7-azabicycloalkenes by tandem olefin metathesis for the preparation of natural product scaffolds**

Nina Deppermann, Marina Büchert, Wolfgang Maison

*General Remarks*: Melting points were determined in open capillaries in a Dr. Lindström instrument and are uncorrected. − ^1^H-NMR and ^13^C-NMR spectra were recorded with a Bruker-Karlsruhe AMX 400 spectrometer (400 MHz / 100.6 MHz) or on a Bruker-Karlsruhe DRX 5001 spectrometer (500 MHz / 125.8 MHz). Chemical shifts, *δ*, are presented in part per million (ppm) and coupling constants, *J*, in Hertz (Hz) from tetramethylsilane (TMS, 0 ppm) as the internal standard for CDCl_3_. – Mass spectra were obtained with a Varian MS MAT 311A in EI mode, a VG/70-250 F (VG Analytical) instrument in FAB mode in a *p*-nitrobenzylalkohol matrix.– The following educts were synthesized according to literature procedures: chiral catalyst **3**[66], azabicycloalkenes **7**[61], **8**[59], **10**[59], **12**[64], pyridinone **9**[59] and isoindoles **11**[59] and **19**[59].

**General procedure 1:** Metathesis of the *N*-acylated azabicycloalkenes

*Method A (with an additive):*

To a 0.01 molar solution of the alkene in abs. DCM 1.1 equiv. 1,4-benzoquinone or 3 equiv. styrene was added. The reaction vessel was evacuated three times using a water jet pump and vented with ethylene from a gas balloon. Catalyst **1** was dissolved in 1 mL abs. DCM and added through a septum. The reaction was heated to reflux or stirred at rt until tlc showed no more conversion. The solvent was evaporated *in vacuo* and the crude product was purified using flash chromatography.

*Method B (without additive):*

A 0.01 molar solution of the alkene in abs. DCM was evacuated three times using a water jet pump and vented with ethylene from a gas balloon. Catalyst **1** was dissolved in 1 mL abs. DCM and added through a septum. The reaction was heated to reflux or stirred at rt until tlc showed no more conversion. The solvent was evaporated *in vacuo* and the crude product was purified using flash chromatography.

**General procedure 2**: Acylation of *N*-Boc protected azabicycloalkenes

A solution of 1 equiv. *N*-Boc-protected amine in 10 mL DCM was heated with 1.2 equiv. triethylamine and 1.1 equiv. iodo-trimethylsilane were added dropwise. After 15 min at reflux, the reaction was cooled to 0°C and 1.3 equiv. methanol were added. After 10 min stirring at 0°C, 1.1 equiv. of the acid chloride were added. The reaction was brought to rt and 20 mL water were added after 1 h. The organic layer was separated and the aqueous layer was extracted with DCM two times. The combined organic layers were dried with Na_2_SO_4_, filtered and the solvent was removed *in vacuo*. The crude product was purified using flash chromatography.

**General procedure 3:** ROM/CM-Metathesis of the *N*-acylated azabicycloalkenes

To a 0.01 molar solution of the azabicycloalkene in abs. DCM and 1.1 to 2.0 equiv. of a terminal alkene was added 3-5 mol% of catalyst **3** and the reaction was stirred under nitrogen atmosphere for 12 h. The solvent was removed *in vacuo* and the crude product was purified using flash chromatography.

*1-(7-Azabicyclo[2.2.1]hept-2-ene-7-yl)-but-3-ene-1-on* ***13***

To a solution of 137 mg azabicycloheptene **12** (1.44 mmol) in 8 mL abs. THF were added 0.13 mL vinylacetic acid (140 mg; 1.60 mmol) and 214 mg HOBt (1.58 mmol). After cooling to 0°C and addition of 326 mg DCC (1.58 mmol) in 3 mL abs. THF the reaction was stirred for 14 h. The precipitate was filtered off and the solvent was removed *in vacuo*. The residue was dissolved in ethyl acetate and washed with sat. NaHCO_3_, sat. KHSO_4_ and brine. The organic layer was dried with Na_2_SO_4_, filtered and the solvent was evaporated *in vacuo*. Flash chromatography of the crude product with petroleum/ethyl acetate 3:1 yielded 145 mg of **13** (0.89 mmol; 61 %) as a colourless wax. ***R*_f_** = 0.40 (PE/EE 1:2; KMnO_4_); **^1^H NMR** (CDCl_3_, 500 MHz, 1:1 mixture of romaters): *δ* = 6.25 - 6.29 (dd, *J* = 6.0 Hz, 2.2 Hz, 1 H), 6.19 - 6.23 (dd, *J* = 6.0 Hz, 2.2 Hz, 1 H), 5.80 - 5.90 (m, 1 H), 5.13 - 5.08 (m, 2 H), 5.07 (bs, 1 H), 4.68 (bs, 1 H), 2.95 - 2.98 (m, 2 H), 1.84 - 1.81 (m, 2 H), 1.18 - 1.25 (m, 1 H), 1.14 - 1.06 (m, 1 H) [ppm]; **^13^C NMR** (CDCl_3_, 100 MHz, mixture of rotamers): *δ* = 166.4, 135.8, 135.6, 134.1, 133.8, 131.4, 118.0, 59.1, 56.6, 39.2, 25.5, 23.1 [ppm]; **HRMS (FAB):** calc. 164.1070 g/mol for C_10_H_13_NO (M + H^+^), found 164.1078 g/mol.

*(3R/S, 8aS/R)-3-Vinyl-2,3,6,8a-tetrahydro-1H-indolizin-5-one* ***14*** *and (3R/S, 8aS/R)-3-Styryl-2,3,6,8a-tetrahydro-1H-indolizin-5-one* ***15***

According to general procedure 1 (method B), 100 mg of alkene **13** (0.63 mmol) was reacted with 46 mg catalyst 1 (0.054 mmol) at rt for 48 h. Flash chromatography of the crude product with petroleum/ethyl acetate 3:1 yielded **14** and **15** in 90% overall yield. **14** was isolated in 81% yield (81 mg) as a yellow oil. ***R*_f_** = 0.19 (PE/EE 1:4); **^1^H NMR** (CDCl_3_, 500 MHz): *δ* = 5.56 (ddd, *J* = 12.3 Hz, 2.8 Hz, 1.3 Hz, 1 H), 5.86 - 5.92 (m, 1 H), 5.85 - 5.90 (m, 1 H), 5.78 (ddd, *J* = 17.0 Hz, 10.1 Hz, 6.6 Hz, 1 H), 5.07 (br, 1 H), 5.01 (br, 1 H); 4.56 (t, *J* = 6.6 Hz, 1 H), 4.08 - 4.16 (m, 1 H), 2.85 - 3.00 (m, 2 H), 2.02 - 2.14 (m, 2 H), 1.67 - 1.83 (m, 2 H) [ppm]; **^13^C NMR** (CDCl_3_, 100 MHz): *δ* = 166.8, 137.5, 127.2, 125.1, 114.5, 59.0, 57.8, 34.5, 30.4, 28.6; **HRMS (FAB):** calc. 164.1070 g/mol for C_10_H_13_NO (M + H^+^), found 164.1075 g/mol (M + H^+^).

**15** was isolated in 9 % yield (13 mg) as a yellow oil. ***R*_f_** = 0.30 (PE/EE 1:4); **^1^H NMR** (CDCl_3_, 500 MHz): *δ* = 7.44 (d, *J* = 7.5 Hz, 2 H), 7.33 (t, *J* = 7.5 Hz, 1 H), 7.22 (t, *J* = 7.5 Hz, 1 H), 6.44 (d, *J* = 11.8 Hz, 1 H), 5.94 – 5.99 (m, 1 H), 5.85 – 5.90 (m, 1 H), 5.59 (dd, *J* = 11.9 Hz, 9.2 Hz, 1 H), 5.01 (t, *J* = 8.8 Hz, 1 H), 4.08 – 4.17 (m, 1 H), 2.92 – 2.97 (m, 2 H), 2.15 – 2.23 (m, 1 H), 2.09 – 2.15 (m, 1 H), 1.86 – 1.91 (m, 1 H), 1.73 – 1.82 (m, 1 H) [ppm]; **^13^C NMR** (CDCl_3_, 100 MHz): *δ* = 161.8, 134.0, 129.2, 128.9, 128.4, 126.9, 126.8, 124.7, 59.4, 54.5, 34.3, 32.1, 30.3, 30.3 [ppm]; **HRMS (FAB):** calc. 240.1388 g/mol for C_16_H_17_NO (M + H^+^), found 240.1366 g/mol (M + H^+^).

According to general procedure 1 (method A), 40 mg of alkene **13** (0.25 mmol) was reacted with 78 mg styrene (0.75 mmol) and 30 mg catalyst **1** (0.035 mmol) at rt for 48 h. Flash chromatography of the crude product with petroleum/ethyl acetate 3:1 yielded **14** and **15** in 81% overall yield. **14** was isolated in 30 % yield (12 mg) as a brown oil. **15** was isolated in 51% yield (30 mg) as a yellow oil.

*1-(7-Azabicyclo(2.2.1)hept-2-ene-7-yl)-pent-4-ene-1-one* ***16***

According to general procedure 2, 0.62 g of azabicycloheptene **12** (3.19 mmol) was reacted with 0.54 mL triethylamine (3.90 mmol), 0.50 mL iodo-trimethylsilane (3.50 mmol), 0.17 mL methanol (4.10 mmol) and 0.39 mL 4-pentenoyl chloride (3.50 mmol). Flash chromatography of the crude product with petroleum/ethyl acetate 4:1 yielded 387 mg of **16** (2.18 mmol; 69%) as a brown oil. ***R*_f_** = 0.26 (PE/EE 2:1; KMnO_4_); **^1^H NMR** (CDCl_3_, 500 MHz): *δ* = 6.28 - 6.32 (dd, *J* = 5.8 Hz, 2.1 Hz, 1 H), 6.20 - 6.25 (dd, *J* = 5.8 Hz 2.1 Hz, 1 H), 5.76 - 5.86 (ddd, *J* = 17.0 Hz, 10.1 Hz, 6.3 Hz, 1 H), 5.09 (bs, 1 H), 5.03 (dd, *J* = 17.0 Hz 1.6 Hz, 1 H), 4.97 (dd, *J* = 10.1 Hz, 1.6 Hz, 1 H), 4.67 (bs, 1 H), 2.27 - 2.36 (m, 2 H), 2.23 - 2.28 (m, 2 H), 1.79 - 1.87 (m, 2 H), 1.18 - 1.25 (m, 1 H), 1.17 - 1.09 (m, 1 H) [ppm]; **^13^C NMR** (CDCl_3_, 100 MHz): *δ* = 166.0, 137.5, 135.8, 133.8, 115.3, 59.1, 56.6, 33.1, 29.2, 25.6, 23.1 [ppm]; **HRMS (FAB):** calc. 178.1226 g/mol for C_11_H_15_NO (M + H^+^), found 178.1232 g/mol (M + H^+^).

*(3R/S,9aS/R)-3-Vinyl-1,2,3,6,7,9a-hexahydro-pyrrolo[1,2-a]azepin-5-one* ***17*** *and (3R/S,9aS/R)-3-Styryl-1,2,3,6,7,9a-hexahydro-pyrrolo[1,2-a]azepin-5-one* ***18***

According to general procedure 1 (method B), 142 mg diene **16** (0.80 mmol) were reacted with 59 mg catalyst **1** (0.07 mmol) at rt for 48 h. Flash chromatography of the crude product with petroleum/ethyl acetate 5:1 yielded **17** and **18** in 73% overall yield. **17** was isolated in 68% yield (96 mg) as a brown oil. ***R*_f_** = 0.21 (PE/EE 1:4) **^1^H NMR** (CDCl_3_, 500 MHz): *δ* = 5.69 – 5.78 (m, 2 H), 5.54 (ddd, *J* = 11.4 Hz, 4.1 Hz, 1.9 Hz), 4.99 – 5.05 (m, 2 H), 4.69 – 4.74 (m, 1 H), 4.48 – 4.55 (m, 1 H), 2.84 – 2.92 (m, 1 H), 1.81 – 1.91 (m, 1 H), 1.71 – 1.80 (m, 1 H), 1.64 – 1.70 (m, 1 H) [ppm]; **^13^C NMR** (CDCl_3_, 100 MHz): *δ* = 173.1, 137.1, 129.7.129.2, 114.2, 59.4, 55.9, 34.5, 33.1, 28.8, 25.3 [ppm]; **HRMS (FAB):** calc. for C_11_H_15_NO 178.1226 g/mol (M + H^+^), found 178.1232 g/mol (M + H^+^).

**18** was isolated in 5 % yield (10 mg) as a brown oil. ***R*_f_** = 0.36 (PE/EE 1:4; UV / KMnO_4_); **^1^H NMR** (CDCl_3_, 500 MHz): *δ* = 7.44 (d, *J* = 7.6 Hz, 2 H), 7.33 (t, *J* = 7.6 Hz, 2 H), 7.22 (t, *J* = 7.6 Hz, 1 H), 6.42 (d, *J* = 11.7 Hz, 1 H), 5.70 - 5.76 (m, 1 H), 5.56 - 5.65 (m, 2 H), 5.15 - 5.21 (m, 1 H), 4.54 - 4.61 (m, 1 H), 2.88 (dt, *J* = 13.3 Hz, 3.5 Hz, 1 H), 2.39 - 2.51 (m, 2 H), 2.24 - 2.37 (m, 2 H), 1.95 - 2.03 (m, 1 H), 1.85 - 1.95 (m, 1 H), 1.75 - 1.83 (m, 1 H) [ppm]; **^13^C NMR** (CDCl_3_, 100 MHz): *δ* = 173.2, 137.0, 133.7, 130.0, 130.0, 129.3, 129.1, 128.4, 127.0, 56.6, 56.1, 34.8, 33.7, 31.1, 25.0 [ppm].

*(2R/S,5S/R)-(E)-tert-Butyl-2-(3-(trimethylsilyl)prop-1-enyl)-5-vinylpyrrolidine-1-carboxylate* ***20***

According to general procedure 3, 40 mg of azabicycloalkene **12** (0.20 mmol) were reacted with 0.04 mL allyltrimethylsilane (0.26 mmol) and 10 mg catalyst **1** (0.012 mmol). Flash chromatography of the crude product with petroleum/ethyl acetate 7:1 yielded a cis/trans-mixture of the product **20** as a brown oil in 97% yield (60 mg). ***R*_f_** = 0.86 (PE/EE 2:1; KMnO_4_); **^1^H NMR** (CDCl_3_, 500 MHz, 1:1 mixture of *cis*/*trans-*isomers): *δ* = 5.70 - 5.84 (br, 1 H), 5.46 - 5.60 (br, 0.5 H), 5.32 - 5.46 (br, 0.5 H), 5.00 - 5.29 (br, 3 H) 4.43 - 4.57 (br, 0.5 H), 4.20 - 4.40 (br, 1.5 H), 1.51 - 2.03 (m, 5 H), 1.41 (br, 10 H), 0.00 (s, 5 H), -0.02 (s, 4 H) [ppm]; **^13^C NMR** (CDCl_3_, 100 MHz, mixture of *cis*/*trans-*isomers): *δ* = 154.8, 140.2, 140.0, 130.3, 129.9, 127.4, 125.9, 114.0, 79.2, 60.6, 55.2, 28.6, 28.6, 18.9, -1.6, -1.9 [ppm]; **MS (FAB):** 310.1 (M + H^+^); **HRMS (FAB):** calc. for C_17_H_32_NO_2_Si 310.2202 g/mol (M + H^+^), found 310.2204 g/mol (M + H^+^).

*(5S/R,11aR/S)-5-Ethyl-5,7,9,10,11,11a-hexahydro-azepino[2,1-a]-isoindole-7-one* ***11***

According to general procedure 1 (method B), 50 mg alkene **10** (0.22 mmol) was reacted with 15 mg catalyst **3** (0.018 mmol) for 48 h at rt. The solvent was evaporated and the crude product was dissolved in 20 mL methanol and stirred with Pd/C under hydrogen atmosphere for 48 h. Flash chromatography of the crude product with petroleum/ethyl acetate 5:1 yielded the product as a colourless solid in 61% yield (31 mg). ***R*_f_** = 0.40 (PE/EE 1:2; UV / KMnO_4_); **Mp.:** 64 °C; **[α]_D_^20^** = -10° (*c* 0.11, CHCl_3_); **HPLC** (CHIRALPAK® AD-H (5 μm), hexanes: *iso*-propanol 9:1; flow 1 ml/min; λ = 210 nm): *ee* = 69 %; *t*_R_ (minor) = 11.5 min; *t*_R_ (major) = 16.5 min; **^1^H NMR** (CDCl_3_, 500 MHz): *δ* = 7.27 - 7.31 (m, 2 H), 7.20 - 7.24 (m, 1 H), 7.15 - 7.19 (m, 1 H), 5.29 (dd, *J* = 6.0 Hz, 2.8 Hz, 1 H), 4.94 (d, *J* = 10.4 Hz, 1 H), 2.64 (dd, *J* = 14.8 Hz, 1 H), 2.50 - 2.58 (m, 1 H), 2.16 - 2.26 (m, 2 H), 2.09 - 2.16 (m, 1 H), 1.92 - 1.99 (m, 2 H), 1.70 - 1.78 (m, 1 H), 1.60 - 1.68 (m, 2 H), 0.75 (t, *J* = 7.4 Hz, 3 H) [ppm]; **^13^C NMR** (CDCl_3_, 100 MHz): *δ* = 174.9, 140.9, 139.6, 128.0, 127.7, 122.7, 122.4, 65.1, 64.2, 38.8, 36.4, 30.2, 26.9, 23.8, 9.1 [ppm]; **HRMS (FAB):** calc. 230.1545 g/mol for C_15_H_20_NO (M + H^+^), found 230.1544 g/mol (M + H^+^).
